# Integrated Genome-Wide Methylation and Expression Analyses Reveal Key Regulators in Osteosarcoma

**DOI:** 10.1155/2020/7067649

**Published:** 2020-08-13

**Authors:** Fei Wang, Guoqing Qin, Junzhi Liu, Xiunan Wang, Baoguo Ye

**Affiliations:** ^1^Department of Orthopedics, China-Japan Union Hospital Jilin University, Changchun, Jilin, China; ^2^Department of Orthopedics, Jilin Disabled Persons' Rehabilitation Center, Jilin Chunguang Rehabilitation Hospital, Changchun, Jilin, China; ^3^Quality Control Department, China-Japan Union Hospital Jilin University, Changchun, Jilin, China; ^4^Department of Orthopedics, The 964th Hospital of the PLA Joint Logistics Support Force, No. 4799 Xi'an Road, Lvyuan District, Changchun City, Jilin Province, China; ^5^Department of Anesthesiology, China-Japan Union Hospital Jilin University, Changchun, Jilin, China

## Abstract

Osteosarcoma (OS) is one of the most common types of primary bone tumors in early adolescence with unsatisfied prognosis. Aberrant DNA methylation had been demonstrated to be related to tumorigenesis and progression of multiple cancers and could serve as the potential biomarkers for the prognosis of human cancers. In conclusion, this study identified 18 downregulated hypomethylation genes and 52 upregulated hypomethylation genes in OS by integrating the analysis the GSE97529 and GSE42572 datasets. Bioinformatics analysis revealed that OS-specific methylated genes were involved in regulating multiple biological processes, including chemical synaptic transmission, transcription, response to drug, and regulating immune response. KEGG pathway analysis showed that OS-specific methylated genes were associated with the regulation of Hippo, cAMP calcium, MAPK, and Wnt signaling pathways. By analyzing R2 datasets, this study showed that the dysregulation of these OS-specific methylated genes was associated with the metastasis-free survival time in patients with OS, including CBLN4, ANKMY1, BZW1, KRTCAP3, GZMB, KRTDAP, LY9, PFKFB2, PTPN22, and CLDN7. This study provided a better understanding of the molecular mechanisms underlying the progression and OS and novel biomarkers for the prognosis of OS.

## 1. Introduction

Osteosarcoma (OS) is one of the most common types of primary bone tumors in early adolescence, which was characterized by an aggressive osteolytic or osteoblastic appearance with a periosteal reaction [1]. Chemotherapy and surgery are the most important treatments for patients with OS [2, 3]. The survival rate of primary OS patients after treatments remains at 60–70% [4]. However, the prognosis of patients with progressive or recurrent OS was less than 20% [5]. In the past decades, emerging studies reported that multiple factors are associated with the tumorigenesis and progression of OS, including germline genetic variants [6], dysregulation of oncogenes or tumor suppressors [7], and the abnormal epigenetics change [8, 9]. A few proteins had been revealed to be related to the progression of OS. For example, GFRA1 was reported to promote autophagy and cisplatin-induced chemoresistance in OS [10]. The isoform 1 of TMIGD3 suppressed OS progression though downregulating NF-*κ*B [11]. Understanding the mechanisms related to OS development could provide new targets for OS.

DNA methylation could affect the gene expression though suppressing transcription [12]. Aberrant DNA methylation had been demonstrated to be involved in regulating tumorigenesis and progression of multiple cancers [13, 14]. In OS, DNA methylation-mediated suppression of miR-449c could promote cell cycle though inhibiting c-Myc in OS [15]. Hypomethylation of IRX1 was found to promote OS metastasis by activating CXCL14/NF-*κ*B signaling [16]. Very interestingly, recent studies showed that aberrant DNA methylation was associated with the prognosis of OS. For example, the DNA methylation level of WNT6 was negatively correlated to the prognosis of children with osteosarcoma [17]. The hypermethylation of ESR1 was correlated to the worse overall survival of OS [18]. These results suggested that the DNA methylation status could be potential diagnostic and therapeutic targets for OS.

The present study analyzed the GSE97529 [19] dataset to identify OS-specific methylated genes. In silico analyses were performed to explore the functions of OS-specific methylated genes. Next, the GSE42572 dataset was used to validate the expression levels of OS-specific methylated genes [20]. Of note, we found that these OS-specific methylated genes were correlated to the prognosis of patients with OS. By these methods, it is hopeful that novel aberrant methylation genes and pathways will be screened in the OS and an understanding of the underlying molecular mechanisms will be enhanced.

## 2. Materials and Methods

### 2.1. Microarray Data

The present study is aimed at identifying dysregulated OS-specific methylated genes in OS by analyzing public databases with bioinformatics analysis. Thus, we screened the GEO databases. The candidate databases were selected according to 3 standards: (1) the candidate database should contain clinical OS samples, (2) the number of clinical samples should be more than 10 cases, and (3) the candidate database was not noncoding RNA datasets. Finally, only the SE97529 and GSE42572 datasets were selected for further analysis. We have included this information in Materials and Methods. The GSE97529 dataset was used to identify OS-specific methylated genes, which was downloaded from the NCBI GEO database (GSE97529). A total of 10 Ewing's sarcoma, 11 synovial sarcoma, and 15 OS samples were included in this dataset. The GSE42572 dataset was analyzed to identify differently expressed genes in OS compared to normal samples, which was also downloaded from the NCBI GEO database (GSE42572). Differentially expressed genes (DEGs) and differentially methylated genes (DMGs) were identified by applying GEO2R. *P* < 0.05 and ∣fold change | ≥2 is set as the cutoff criterion.

### 2.2. Functional and Pathway Enrichment Analyses

The DAVID system was used to predict the potential biological processes and KEGG pathways involved in target genes in this study [21]. *P* < 0.05 was set as the cutoff criterion.

### 2.3. Protein-Protein Interaction (PPI) Network Analysis

In the present study, PPI networks were used to reveal the interactions among differentially expressed OS-specific methylated genes using the STRING database (https://string-db.org/). PPI was visualized using Cytoscape [22].

### 2.4. Survival Analysis

Survival analysis was performed using the OS microarray dataset (mixed osteosarcoma (mesenchymal)-Kuijjer-127-vst-ilmnhwg6v2) from the R2: Genomics Analysis and Visualization Platform (http://r2.amc.nl). The median expression of targets was selected as the cutoff to divide all OS samples into the high or low group.

## 3. Results

### 3.1. Identification of OS-Specific Methylated Genes

**Figure 1:**
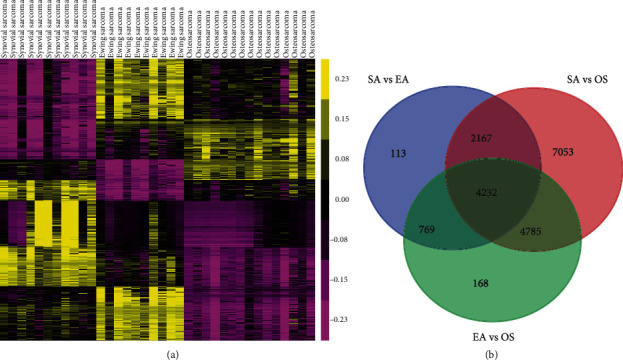
OS-specific methylated genes were identified by using the public dataset GSE97529. (a) DNA methylation status of 482,421 CpG sites in 10 Ewing's sarcoma, 11 synovial sarcoma, and 15 OS samples were included in this dataset.

The public dataset GSE97529 was used to identify OS-specific methylated genes. DNA methylation status of 482,421 CpG sites in 10 Ewing's sarcoma, 11 synovial sarcoma, and 15 OS samples were included in this dataset (Figure 1(a)). Totally, we identified 3125 OS-specific methylated genes, including 875 hypermethylation genes and 2250 hypomethylation genes in OS samples compared to Ewing's sarcoma or synovial sarcoma samples (Figure 1(a)).

### 3.2. GO and KEGG Pathway Enrichment Analyses

**Figure 2:**
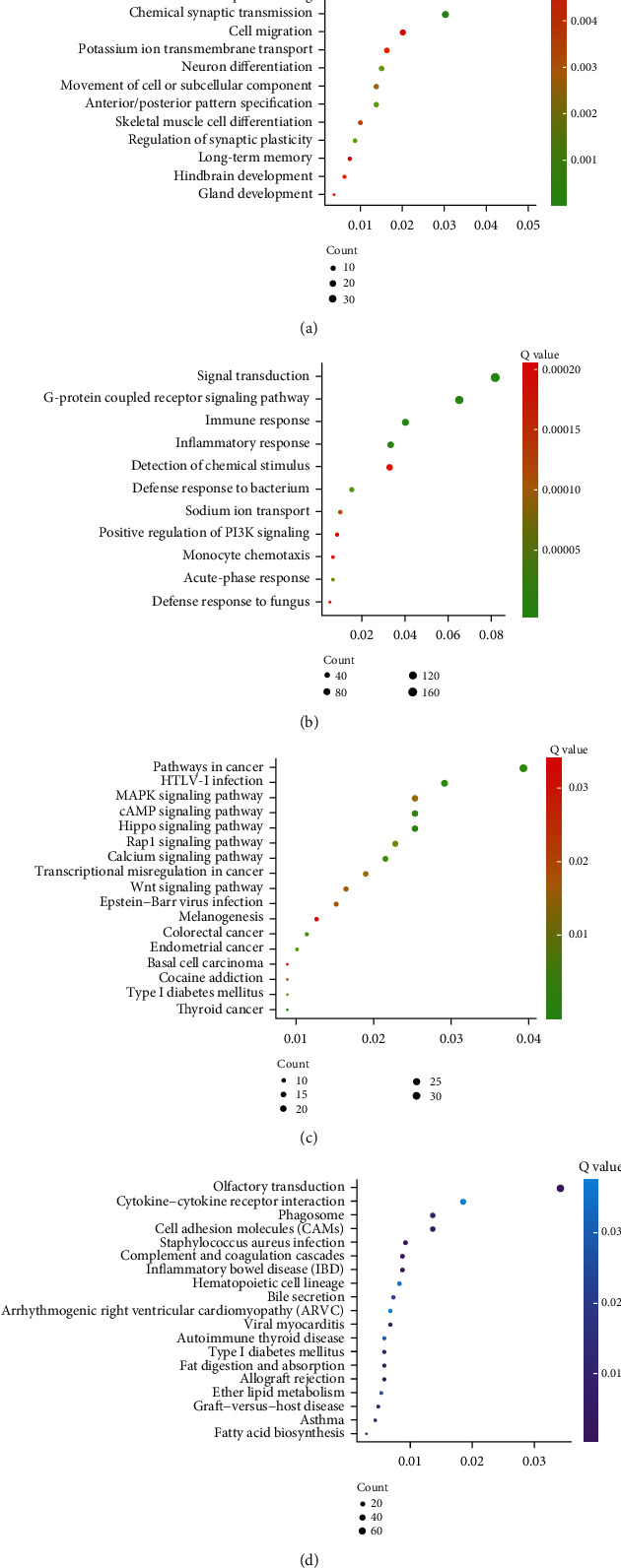
Bioinformatics analysis of hypermethylation genes and hypomethylation genes. (a) GO analysis of OS-specific hypermethylation genes. (b) GO analysis of OS-specific hypomethylation genes. (c) KEGG pathway analysis of OS-specific hypermethylation genes. (d) KEGG pathway analysis of OS-specific hypomethylation genes. The gene ratio was present in the *X*-axis.

GO analysis showed that hypermethylation genes were significantly associated with biological processes (BP) of the nervous system development, chemical synaptic transmission, transcription from RNA polymerase II promoter, anterior/posterior pattern specification, regulation of synaptic plasticity, neuron differentiation, movement of cell or subcellular component, skeletal muscle cell differentiation, response to drug, potassium ion transmembrane transport, hindbrain development, gland development, and cell migration (Figure 2(a)). Hypomethylation genes were significantly related to immune response, signal transduction, inflammatory response, acute-phase response, sodium ion transport, monocyte chemotaxis, detection of chemical stimulus, defense response to fungus, positive regulation of PI3K pathway, cell chemotaxis, chemotaxis, neutrophil chemotaxis, innate immune response, ion transmembrane transport, and cell adhesion (Figure 2(b)).

KEGG pathway analysis showed that significant pathways of hypermethylation genes in OS included the Hippo pathway, cAMP signaling, thyroid cancer, pathways in cancer, calcium signaling, endometrial cancer, Rap1 signaling pathway, transcriptional misregulation in cancer, MAPK signaling pathway, Epstein-Barr virus infection, Wnt signaling pathway, cocaine addiction, and basal cell carcinoma (Figure 2(c)). And hypomethylation genes in OS were associated with Staphylococcus aureus infection, olfactory transduction, inflammatory bowel disease (IBD), complement and coagulation cascades, allograft rejection, fat digestion and absorption, graft-versus-host disease, phagosome, viral myocarditis, and fatty acid biosynthesis (Figure 2(d)).

### 3.3. OS-Specific Methylated Genes Were Differentially Expressed in OS

**Figure 3:**
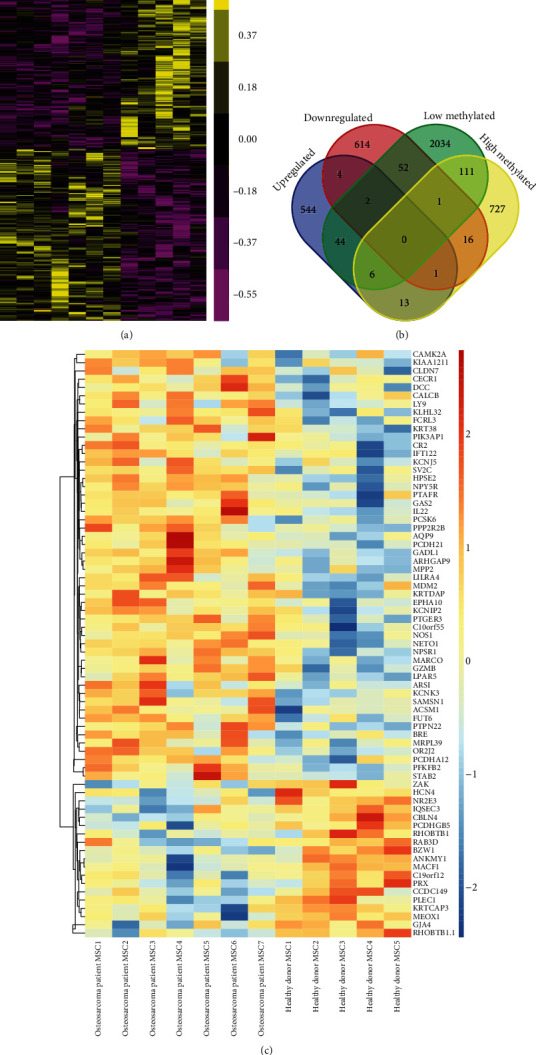
GSE42572 was analyzed to identify differently expressed genes in OS compared to normal samples. (a) 614 induced genes and 696 reduced genes in OS compared to healthy control samples. (b) Among DEGs, a total of 18 downregulated hypomethylation genes and 52 upregulated hypomethylation genes were screened out. (c) The 70 differentially expressed OS-specific methylated genes were presented by heat map.

Subsequently, an independent public dataset, GSE42572, was used to identify differentially expressed genes in OS. As shown in Figure 3(a), we identified 614 upregulated genes and 696 downregulated genes in OS compared to healthy control samples (Figure 3(a)). Among DEGs, a total of 18 downregulated hypomethylation genes were screened out from overlapping 875 hypermethylation and 690 downregulated genes, while 52 upregulated hypomethylation genes were screened out from overlapping 2250 hypomethylation and 614 downregulated genes (Figure 3(b)). The 70 differentially expressed OS-specific methylated genes were presented by heat map (Figure 3(c)).

### 3.4. Construction of PPI Network to Identify Hub Differentially Expressed OS-Specific Methylated Genes.

**Figure 4:**
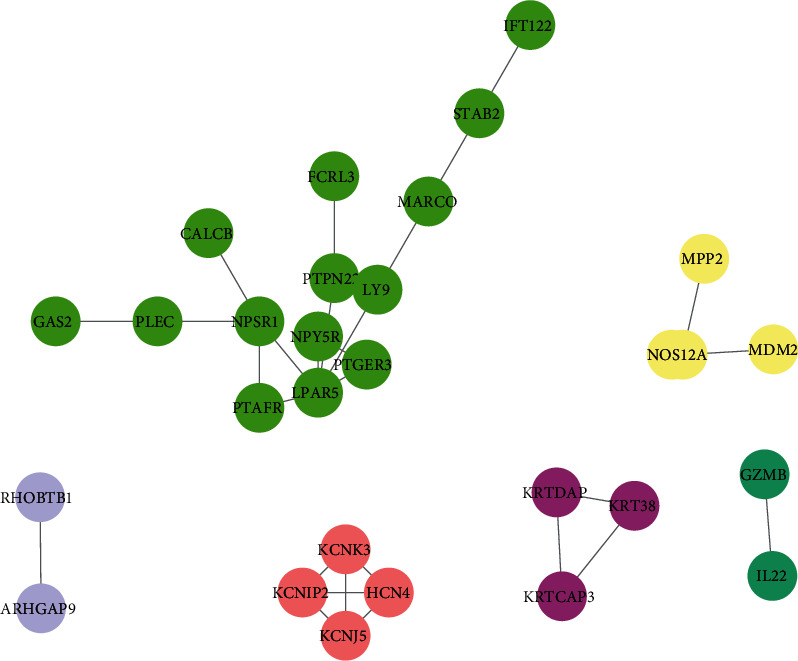
PPI network analysis. PPI network was used to identify a hub differentially expressed OS-specific methylated gene using the STRING database.

Furthermore, we constructed a PPI network to identify a hub differentially expressed OS-specific methylated gene using the STRING database. As presented in Figure 4, a total of 29 nodes and 30 edges were included in this network. The hub genes included NPSR1, PTAFR, LPAR5, PTGER3, NPY5R, KCNK3, KRTDAP, HCN4, KRT38, KCNIP2, KCNJ5, and KRTCAP3 (Figure 4).

### 3.5. The Survival Time Analysis of Differentially Expressed OS-Specific Methylated Genes

The above analysis was conducted with the GSE97529 and GSE42572 datasets. Unfortunately, the clinical information about metastasis-free survival time was not included in both databases. Thus, we analyzed an independent database, R2 dataset (http://r2.amc.nl), to further evaluate the prognostic value of OS-specific methylated genes. The median expression of candidates in all OS samples was selected.

**Figure 5:**
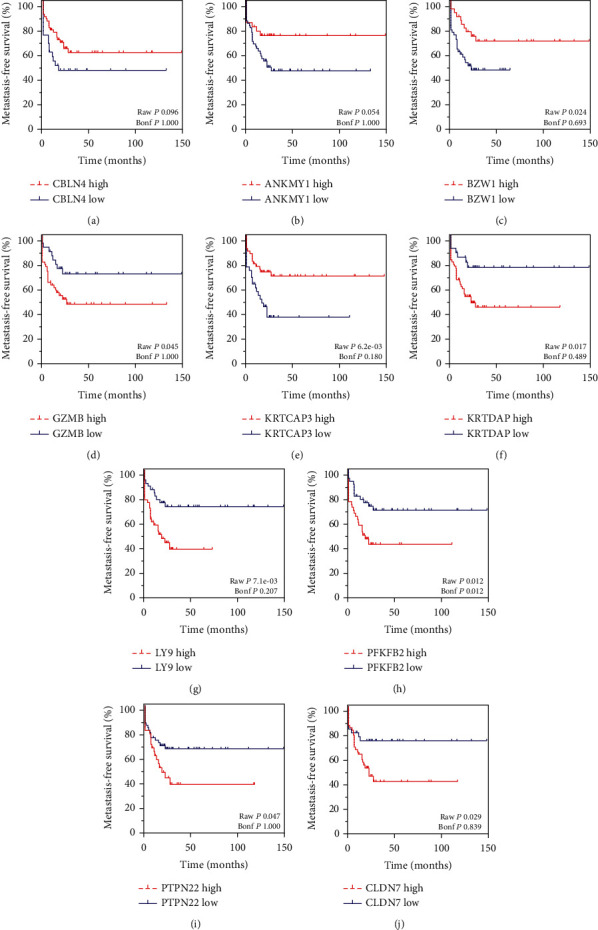
The prognostic values of differentially expressed OS-specific methylated genes were calculated by using the R2: Genomics Analysis and Visualization Platform. (a–j) Higher expression of CBLN4 (a) was associated with longer metastasis-free survival time in patients with OS, as well as ANKMY1 (b), BZW1 (c), and KRTCAP3 (e). However, higher expression of GZMB (d), KRTDAP (f), LY9 (g), PFKFB2 (h), PTPN22 (i), and CLDN7 (j) was associated with shorter metastasis-free survival time in patients with OS.

As the cutoff is used to divide OS samples into the high and low groups, it was shown that higher expression of CBLN4 (*P* < 0.05) was associated with longer metastasis-free survival time in patients with OS, as well as ANKMY1 (*P* < 0.05), BZW1 (*P* < 0.05), and KRTCAP3 (*P* < 0.001). However, higher expression of GZMB (*P* < 0.05), KRTDAP (*P* < 0.05), LY9 (*P* < 0.05), PFKFB2 (*P* < 0.05), PTPN22 (*P* < 0.05), and CLDN7 (*P* < 0.05) was associated with shorter metastasis-free survival time in patients with OS (Figure 5).

## 4. Discussion

The mechanisms underlying OS progression remained largely unclear. It has been widely accepted that DNA methylation was involved in regulating the tumorigenesis and development though modulating gene expression. DNA methylation has been shown to play an important role in gene regulation and implicated in various types of cancer. Emerging studies revealed that the cancer-specific CpG hypermethylation could turn off the expression of tumor suppressors; however, cancer-specific CpG hypomethylation could activate the expression of oncogenes [23]. Identification of aberrantly methylated genes in OS would be helpful to identify new diagnostic and therapeutic biomarkers for OS. The present study identified OS-specific methylated genes from Ewing's sarcoma or synovial sarcoma samples. Bioinformatics analysis revealed that OS-specific methylated genes were involved in regulating multiple biological processes, including chemical synaptic transmission, transcription, response to drug, and regulating immune response. Further validation indicated that OS-specific methylated genes were dysregulated in OS samples and correlated to the prognosis of patients with OS.

OS, together with Ewing's Sarcoma (EWS) and synovial sarcoma (SS), was the most common pediatric sarcomas [24]. These types of sarcomas occur in similar anatomical locations; however, the treatments for these sarcomas differed depending on the tumor type. The accurate diagnosis of OS remained to be a big challenge. Emerging studies demonstrated that aberrant DNA methylation was associated with the prognosis of human cancers, including OS. For example, DNA methylation level of WNT6 and ESR1 was related to the prognosis of OS. The present study is aimed at identifying OS-specific methylated genes. A total of 3125 OS-specific methylated genes were identified, including 875 hypermethylation genes and 2250 hypomethylation genes in OS samples compared to Ewing's sarcoma or synovial sarcoma samples. Furthermore, GO and KEGG pathway analyses were further used to predict the potential roles of OS-specific methylated genes. Of note, our predictions showed that these methylated genes were associated with the Hippo signaling and Wnt signaling. Hippo pathway aberrations had been demonstrated in OS by multiple studies and involved in regulating primary tumor growth, angiogenesis, epithelial to mesenchymal transition, and metastatic dissemination [25]. The Hippo signaling played an important role controlling cancer cell proliferation and apoptosis [26]. Multiple studies indicated YAP was overexpressed in OS samples, and knockdown of YAP significantly inhibits OS cell growth and invasion [27]. Sox2, as a YAP upstream regulator, was reported to be required for tumor development and cancer cell proliferation in OS [28]. This study provided a potential mechanism to elucidate how the Hippo signaling activated in OS. Many studies support an aberrant activation of the canonical Wnt signaling pathway in osteosarcoma cells. For example, two recent studies described a high *β*-catenin level in osteosarcoma tissues compared to adjacent healthy tissues associated with poor prognosis and lung metastatic dissemination. Wnt signaling pathway played a crucial role in tumorigenicity and metastasis via regulation of the immune system, bone remodeling, angiogenesis, hypoxia response, and EMT [29].

Of note, this study showed that OS-specific methylated genes were significantly differentially expressed in OS samples. A total of 18 downregulated hypomethylation genes and 52 upregulated hypomethylation genes were identified in this study. PPI network analysis was constructed to reveal the relation among these genes. Totally, 29 nodes and 30 edges were included in this network. By analyzing R2 datasets, we found the dysregulation of these OS-specific methylated genes were associated with the metastasis-free survival time in patients with OS, including CBLN4, ANKMY1, BZW1, KRTCAP3, GZMB, KRTDAP, LY9, PFKFB2, PTPN22, and CLDN7. Among these regulators, BZW1 is a transcription factor related to the regulation of cell cycle and proliferation [30]. LY9 was a member of SLAM family of immunomodulatory receptors [31] and interacted with the adaptor molecule signaling lymphocyte activation molecule-associated proteins. A previous study showed LY9 was related to the cancer progression and correlated to overall survival of the patients with breast cancer. PFKFB2 is an enzyme involved in regulating the Warburg effect (also termed as glycolysis) [32]. PFKFB2 had been found to have a key role in regulating tumor growth and survival in multiple cancer types, including gastric cancer, gliomas, and osteosarcoma [32–37].

Several limitations were also exited in this study. First, our studies revealed several hub OS-specific methylated genes. However, the roles of these genes remained to be unclear. The gain or loss of function assays should be performed to further explore their roles in OS. Next, the expression levels and methylation levels of hub OS-specific methylated genes in OS samples should be confirmed using clinical samples. Third, the direct interaction among these hub genes has not been confirmed using experimental assays.

## 5. Conclusion

In conclusion, this study identified 18 downregulated hypomethylation genes and 52 upregulated hypomethylation genes in OS and a series biological processes and pathways regulated by aberrantly methylated genes. PPI network analysis revealed the interactions among these genes. Moreover, the present study showed that the dysregulation of OS-specific methylated genes was correlated with the metastasis-free time in patients with OS, including CBLN4, ANKMY1, BZW1, KRTCAP3, GZMB, KRTDAP, LY9, PFKFB2, PTPN22, and CLDN7. This study provided a better understanding of the molecular mechanisms underlying the progression and OS and novel biomarkers for the prognosis of OS.

## Data Availability

The data used to support the findings of this study are available from the corresponding author upon request.
